# Symptom Clusters in Acute SARS-CoV-2 Infection and Long COVID Fatigue in Male and Female Outpatients

**DOI:** 10.3390/jpm14060602

**Published:** 2024-06-05

**Authors:** Vincenza Leone, Dennis Freuer, Yvonne Goßlau, Inge Kirchberger, Tobias Warm, Alexander Hyhlik-Dürr, Christine Meisinger, Jakob Linseisen

**Affiliations:** 1 Epidemiology, Medical Faculty, University of Augsburg, 86156 Augsburg, Germany; 2 Clinic for Vascular Surgery, Medical Faculty, University Hospital, 86156 Augsburg, Germany; 3 Institute for Medical Information Processing, Biometry and Epidemiology—IBE, Marchionistraße 15, 81377 Munich, Germany

**Keywords:** SARS-CoV-2, Post-COVID-19, Long COVID, fatigue, acute COVID-19 symptom clusters, outpatients

## Abstract

(1) Background: After an acute SARS-CoV-2 infection, patients are at risk of developing Long COVID, with fatigue as a frequent and serious health problem. Objectives: To identify symptom clusters in acute SARS-CoV-2 infections and investigate their associations with the development of Long COVID fatigue, and to examine sex-specific differences. (2) Methods: The analysis included a total of 450 COVID-19 outpatients, of whom 54.4% were female. The median ages of the men and women were 51 years (IQR 36.0; 60.0) and 48 years (IQR 33.0; 57.0), respectively. Data collection took place between November 2020 and May 2021, with a median time between acute SARS-CoV-2 infection and examination in the study center of 240 days (IQR 133; 326). The Fatigue Assessment Scale (FAS) was used to identify fatigue and its severity. A multiple correspondence analysis was used to group forty-two COVID-19 symptoms into seven symptom clusters. Logistic and log-linear regressions were used to investigate associations between acute symptom clusters and Long COVID fatigue as dichotomous and continuous outcome, respectively. (3) Results: Fatigue occurred more frequently in women than in men (45% vs. 25%) and the median FAS score, indicating severity of fatigue, was higher in women than in men. The comparison between men and women revealed notable differences in four out of seven clusters. The strongest associations between symptom clusters in infection and Long COVID fatigue were identified for the cluster “cognitive and mental symptoms”. In the log-linear regression model, each additional symptom in this cluster was associated with an increase of the FAS score by 5.13% (95% CI: [0.04; 0.07]; *p* < 0.001). The results of the logistic regression models supported this finding. Each additional symptom in this symptom cluster increased the odds of fatigue by 42% (95% CI: [1.23; 1.66]; *p* < 0.001). (4) Conclusions: In our study in COVID-19 outpatients, a strong association was observed between the number of symptoms in the cluster “cognitive and mental symptoms” during acute SARS-CoV-2 infection and the risk of developing fatigue months later. The consequent use of preventive and therapeutic strategies is necessary to decrease the burden of fatigue in the context of Long COVID.

## 1. Introduction

After an acute SARS-CoV-2 infection, long-lasting complaints and symptoms may occur, which is usually referred to as Long COVID or Post-COVID syndrome. Long COVID is classified as experiencing persistent symptoms ≥ 4 weeks post-infection [1], and we will use this term in this manuscript. Long COVID is present when (1) the infected person still suffers from ongoing or new symptoms after the SARS-CoV-2 infection or its treatment; (2) new symptoms which can be understood as a consequence of a SARS-CoV-2 infection occur after the end of the acute phase; and (3) a pre-existing condition is worsening as a result of a SARS-CoV-2 infection [1]. The published prevalence data on Long COVID vary widely due to differences in size and type of patient groups (e.g., hospitalization status), symptoms assessed (e.g., self-reported vs. clinically diagnosed), time of the anamnesis, and type of laboratory test for the SARS-CoV-2 diagnosis (self-report, PCR test, serologic test). As diagnostic methods may either detect active infections or identify past infections, the choice of the method can influence the number of diagnosed COVID-19 cases, and thus the prevalence of Long COVID [1,2]. O’Mahoney et al. conducted a systematic review and meta-analysis and estimated an average of 45% of SARS-CoV-2 infected patients suffering from Long COVID [3]. One of the most common symptoms of Long COVID is fatigue [1,3,4,5]. Fatigue is classified as one of the core symptoms of the chronic fatigue syndrome (CFS), also known as myalgic encephalomyelitis (ME) [6]. CFS/ME often occurs after an acute infection caused by various pathogens, such as the Epstein–Barr virus and influenza virus. Chronic viral infections can lead to fatigue by causing persistent inflammation, demyelination, and immune cell activation, which result in neuroinflammatory responses and muscle weakness [7,8]. Some individuals developed CFS/ME after the SARS pandemic in 2002/2003, and a subgroup of COVID-19 patients shows similar symptoms [9,10]. Fatigue in CFS/ME is characterized by flu-like symptoms, especially at the onset of the illness, a restlessness accompanied by feeling both wired and tired, low energy or lack of physical strength to initiate or complete daily activities, cognitive fatigue exacerbating existing difficulties, and a rapid loss of muscle strength or endurance after initiating an activity, resulting in sudden weakness, clumsiness, lack of coordination, and the inability to sustain physical efforts consistently [11]. With respect to Long COVID fatigue, some studies reported that women are more likely to be affected [1,12,13], whereas a meta-analysis by Ceban et al. reported a proportion of 32% (95% CI: [27%; 37%]; *p* < 0.001; N = 25,268) with fatigue after COVID-19, with no significant sex difference [14]. These differences could be due to different study populations, especially the differences in disease severity. Ceban et al. included participants with severe courses (hospitalized patients and patients treated in the intensive care unit) in their meta-analysis [14]. Another relevant aspect could be the differences in data collection time points during the COVID-19 pandemic, as different SARS-CoV-2 mutations spread over the course of the pandemic [15]. Since the data available in the literature cannot clearly confirm whether there are sex differences regarding fatigue in Long COVID, we aim to investigate this again with our analysis. Furthermore, chronic fatigue represents a financial burden for the economic and health care system, as well as a burden for the patients themselves and their families, especially because no effective therapy has been established yet [16]. So far, it is unclear whether there is an association between the severity of acute SARS-CoV-2 infection and the risk of Long COVID fatigue [1]. Only a few studies investigated the situation in outpatients [3]. An earlier report from our group found an association between the number of symptoms in acute infection and the risk of Long COVID fatigue in outpatients [17]. SARS-CoV-2 symptoms can be clustered into distinct symptom clusters, and these clusters are differently associated with fatigue. In the present paper, we investigated whether symptom patterns or clusters in acute infections are associated with the development of Long COVID fatigue, also addressing sex-specific differences.

## 2. Methods

### 2.1. Study Design

The present analysis is based on data from the COVID Thrombosis (COVID-T) study, a monocentric, cross-sectional, observational study. Patients were recruited by the health departments of the city of Augsburg and Augsburg county. All residents who were registered with a positive SARS-CoV-2 smear until November 2020 (*n* = 1600) received a postal invitation to participate in the study. Eligible participants had to be at least 18 years old and have to be tested positive for SARS-CoV-2 (smear test) at least 14 days prior the study center visit, and without a request for quarantine.

#### 2.1.1. Data Collection

Data collection took place between November 2020 and May 2021 at the University Hospital Augsburg. At the time of data collection, a vaccine against COVID-19 was already approved in Germany, as the first vaccinations were given in December 2020. Participants answered standardized questionnaires, including information on demographics, smoking status, and previous diseases, as well as questions on mental and physical condition, including fatigue. Overall, they were asked forty-two possible symptoms during the acute SARS-CoV-2 infection. The self-administered questionnaire was made available digitally via a tablet; the participants always had the option to check an “I don’t know” box.

#### 2.1.2. Fatigue Assessment

To measure fatigue, the Fatigue Assessment Scale (FAS) [18,19] was used. This questionnaire consists of 10 items, each with five possible answers, ranging from “never” to “always.” score ranging between 10 and 50 points was calculated. Participants with a FAS score of ≥22 were considered to suffer from fatigue [20].

### 2.2. Statistical Analysis

All participants with less than 30 days between acute infection and interview were excluded from the analyses. Regarding diagnoses of depression and anxiety, as well as questions about symptoms during acute SARS-CoV-2 infection, indications of “I don’t know” were coded as “no”. The time interval between examination date and acute SARS-CoV-2 infection was estimated by calculating the difference in days of the examination date and the date of the first positive SARS-CoV-2 smear test. The body mass index (BMI) of the participants was calculated by measured body weight (in kg) divided by height squared (in meters). The medians and interquartile ranges (IQRs) were calculated for all continuous variables. Categorical variables were presented as absolute frequencies and proportions (in percentage). All descriptive data were presented, stratified by sex. Differences between groups were tested using the Wilcoxon rank sum test and Pearson’s Chi-squared test. *p*-values < 0.05 indicated significant results.

Multiple correspondence analysis was used to group COVID-19 symptoms into clusters. Multivariable regression models were performed to examine the associations between each symptom cluster (independent variables) and both the continuous FAS score and the outcome fatigue (yes/no) as dependent variables. All regression models were adjusted for the following possible confounders: age, sex, BMI, smoking status and former diagnoses of depression or anxiety. The FAS score was log-transformed to ensure the assumption of normally distributed residuals in multivariable linear models. For better interpretation, the estimates were converted using the following formula: $\;{(e}^{\beta }-1)\;\times \;100$, and indicate the percentage change in the FAS score. Multivariable logistic regression models were built, and odds ratios (ORs) and 95% confidence intervals (CIs) were calculated to explore associations with fatigue as outcome. Finally, two more mutually adjusted models were performed, including all symptom clusters as determinants of the FAS score (severity of fatigue), or fatigue as a dichotomous (yes/no) outcome. To assess sex-specific effects, all models were additionally stratified by sex. However, the interaction terms with sex were never statistically significant. Regarding multiple testing issues, the *p*-values from regression models were corrected for false discovery rate (FDR) (5%) and labeled as q-values afterwards. All statistical analyses were computed with the statistical software R (version 4.2.1).

## 3. Results

The analysis included 450 outpatients, of whom 54.4% were female. Descriptive statistics are presented in Table 1. The median time between acute SARS-CoV-2 infection and interview/examination was 244 (IQR 143; 322) and 238 (IQR 123; 331) days for men and women, respectively (overall median time: 240 days (IQR 133; 326)). The median ages of the men and women were 51 years (IQR 36.0; 60.0) and 48 years (IQR 33.0; 57.0), respectively. Women showed a significantly higher FAS score (median 20 (IQR 16; 26)) than men (median 18 (IQR 15; 21)) and a significantly higher prevalence of fatigue: almost 45% of the women and nearly 25% of the men suffered from fatigue (*p* < 0.001). Men reported a significantly better mental and physical health than women as assessed with the VR-12 questionnaire.

Based on forty-two symptoms during acute SARS-CoV-2 infection, the multiple correspondence analysis identified seven clusters representing impairment of specific body regions or body functions (Table 2). The cluster variables represent summary scores; for each given symptom in the respective cluster, the score increased by one point. A comparison between men and women revealed notable differences in four out of seven clusters (Table 1).

**Figure 1:**
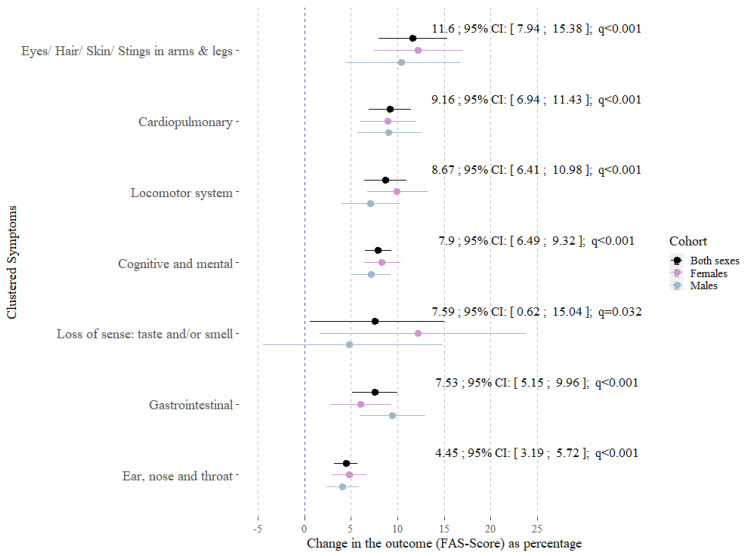
Point estimates, 95% confidence intervals and q-values from multivariable log-linear regression models representing the associations between seven symptom clusters and the FAS score for both sexes. All models were adjusted for age, sex (except sex-specific models), BMI, smoking status and the history of depression or anxiety, (N_Both sexes_ = 446; N_Females_ = 245; N_Males_ = 201).

Figure 1 shows the results from multivariable log-linear regression models estimating the associations between symptom clusters and the FAS score. All clusters were positively associated with the FAS score (Figure 1). The strongest association, with a 11.6% change (95% CI: [7.9; 15.4]; q < 0.001) in the FAS score per additional symptom, was observed in the group of participants with symptoms concerning “eyes, hair, skin and stings in arms or legs”. The weakest associations regarding the point estimate and the precision were observed for symptoms concerning the cluster “ear, nose and throat” and the cluster “Loss of sense: taste and/or smell”.

**Figure 2:**
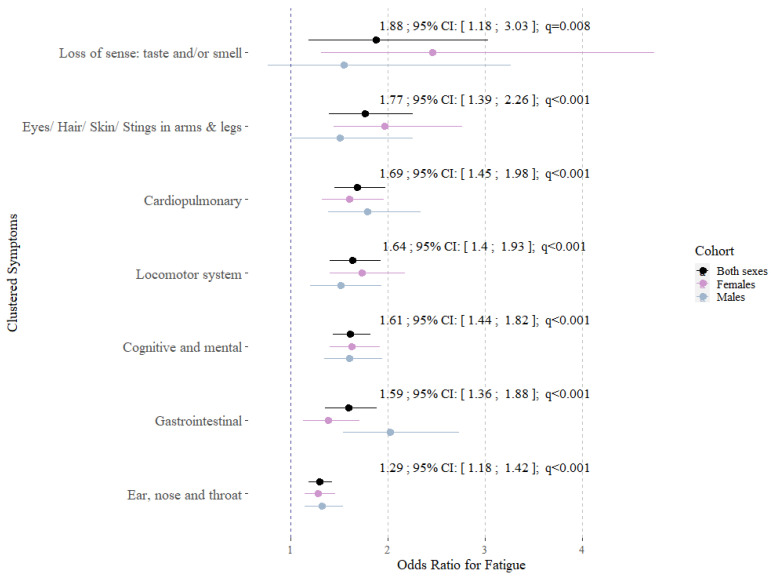
Odds ratios, 95% confidence intervals and q-values from multivariable logistic regression models representing the associations between seven symptom clusters and the development of Long/Post-COVID fatigue for both sexes. All models were adjusted for age, sex (except sex-specific models), BMI, smoking status and the history of depression or anxiety, (N_Both sexes_ = 446; N_Females_ = 245; N_Males_ = 201).

The results were fully confirmed by the multivariable logistic regression models investigating the associations with fatigue (Figure 2).

Table 3 presents the results of the mutually adjusted log-linear regression model, in which all seven cluster variables were included. As a result, only the cluster “cognitive and mental symptoms during acute SARS-CoV-2 infection” was positively associated with the FAS score. For each additional symptom in this cluster, the FAS score increased by 5.13% (95% CI: [7.9; 15.4]; q < 0.001). In sex-specific models, the cluster “cognitive and mental symptoms” was associated with the FAS score in both sexes (men: 5.13% change per additional symptom, (95% CI: [0.02; 0.08]; q = 0.009); women: 6.18% change per additional symptom (95% CI: [0.03; 0.08]; q < 0.001)) (Supplementary Tables S1 and S2).

Table 4 presents the results of the logistic regression model, examining the associations between the mutually adjusted symptoms clusters and the risk of fatigue. The OR for the cluster “cognitive and mental symptoms” during acute SARS-CoV-2 infection remained significant after FDR adjustment. With each additional cognitive or mental symptom, the odds of later fatigue increased by 42% (95% CI: [1.23; 1.66]; q < 0.001). After stratifying the models by sex (N_male_ = 201, N_female_ = 245), the result only remained significant in women (OR 1.45; 95% CI: [1.20; 1.76]; q = 0.002) (Supplementary Tables S3 and S4). Interaction terms with sex were included in all models, but these never reached statistical significance.

## 4. Discussion

In this study, we observed that women are more likely to experience fatigue in Long COVID than men, which aligns with known findings [21,22,23]. Further we identified seven COVID-19 symptom clusters, which were all associated with the risk and severity of Long COVID fatigue. After mutual adjustment, i.e., including all symptom clusters either in a log-linear or a logistic regression model, only the cluster “cognitive and mental symptoms” significantly increased the FAS score (severity of fatigue) and showed an increased risk of fatigue.

Compared to our findings (35.8% suffering from fatigue, with an average of 240 days since infection), other studies have reported higher percentages of individuals experiencing fatigue after COVID-19. Bell et al. found 58% of outpatients experienced fatigue for at least 60 days following acute SARS-CoV-2 infection [24]. Stavem et al. examined non-hospitalized participants, of whom 46% were still experiencing fatigue 4 months after the acute infection [12], and Lombardo et al. surveyed patients 12 months after acute infection, and found fatigue in 52%. However, Lombardo et al. made no stratification regarding hospitalization status, which could explain the higher percentage of patients experiencing fatigue one year after the acute infection [25]. In line with findings from other studies [12,13,25], and also in our study, more women experienced fatigue COVID-19 compared to men.

No study was published which could be directly compared with our results, i.e., the type of symptom clusters formed and their associations with later fatigue.

Huang et al. [26] have chosen a similar methodological approach to establish symptom clusters, though based on a distinctly lower number of symptoms. Symptoms during the acute infection and 180 days after the infection were clustered, but these clusters were not modelled specifically for their association with fatigue. However, in a cohort study of hospitalized SARS-CoV-2 patients (*n* = 1969), Fernandez et al. found that the two patient clusters with a higher number of symptoms during acute infection were more likely to suffer from later fatigue. Fatigue was assessed here at a median of 8.4 months post infection, which is comparable to the present study [27]. The finding that a higher number of symptoms during SARS-CoV-2 infection is more likely to result in later fatigue is also confirmed by the results of Stavem et al., who examined non-hospitalized patients (*n* = 458), who answered a questionnaire at a median of 117.5 days after acute infection [12]. Results from our previous work confirmed these findings: outpatients in the Long COVID fatigue group had a higher median number of acute SARS-CoV-2 symptoms [17]. Finally, Whitaker et al. formed clusters at the onset of symptoms, and re-clustered the symptoms assessed 84 days after acute SARS-CoV-2 infection. However, it cannot be determined which initial symptoms contribute to fatigue or tiredness later in time. Participants who suffered from later tiredness or later severe fatigue initially more frequently reported shortness of breath or muscle aches [28]. Our results confirm the initial symptoms of “tiredness or exhaustion” and sleepiness, which are grouped to the cluster “cognitive and mental”, which is associated with later fatigue in our study. Since Whitaker et al. only formed two clusters, a direct comparison with the results of our seven clusters analysis is not possible.

Dixon et al. performed an exploratory factor analysis, clustering acute SARS-CoV-2 symptoms, including *n* = 8214 randomly selected individuals. However, they used only 14 symptoms, though the grouping showed similarities to our symptom clusters. The differences were that the symptom of loss of taste and smell was grouped with the symptom of fever [29]. Sudre et al. summarized symptoms reported by 1653 participants, of whom 383 reported at least one hospital visit. The occurrence of 14 symptoms was clustered by the median duration of symptoms into six clusters. However, symptoms could appear simultaneously in different clusters due to another type of clustering methodology [30]. Nevertheless, we found parallels with two clusters that summarized milder forms of the SARS-CoV-2 infection according to Sudre et al. These clusters grouped the symptoms cough, fever, headache and sore throat [30], as we did in our “ear, nose and throat” cluster. In contrast to our analyses, these clusters also contained the symptom “loss of smell”, which we grouped differently. Molina-Mora et al. analyzed data from *n* = 18,974 participants to identify seven symptom clusters during acute SARS-CoV-2 infection, as well as the frequency of SARS-CoV-2 PANGOLIN lineages within the clusters. Similar to Sudre et al., the colleagues included a lower number of symptoms (*n* = 18) in their cluster analysis, and symptoms could occur simultaneously in each cluster. The frequency of symptoms are visualized as a heat map per cluster. The results of the colleagues confirm our result of the symptom cluster “Loss of sense: taste and/or smell” [31]. The other clusters show higher frequencies of the symptoms cough, fever and headache, which shows a similar symptom clustering to Sudre et al. and our cluster “Ear, nose and throat” [30]. However, the cluster analysis by Molina-Mora et al. showed a low frequency of the symptom sore throat across all clusters [31]. During the time of data collection, the SARS-CoV-2 variants Alpha (B.1.1.7), Beta (B.1.351), Gamma (P.1) and Delta (B.1.617.2) predominated in Germany [32]. Molina-Mora et al. found no association between the clusters and the PANGOLIN lineage. However, when looking at the distribution of the measured PANGOLIN lineages across the clusters, B.1 and B.1.1.389 are more frequently represented than others [31]. These do not correspond to the predominant PANGOLIN lineages in Germany at the time and are not among the variants of concern defined by the World Health Organization [15].

The findings in our study, suggesting an association between “cognitive and mental symptoms” during acute COVID-19 infection and later fatigue (in the context of Long COVID), have important clinical implications. This cluster includes the symptoms tiredness or exhaustion, sleepiness, concentration difficulties, sleep disorders, memory problems, depressed mood, anxiety or panic, mood swings. Our findings underscore the need for the early identification and targeted monitoring of patients exhibiting these symptoms to identify those at risk of developing Long COVID fatigue. Secondly, the results highlight the potential for intervention strategies aimed at reducing the severity or duration of these symptoms during acute infection, thus mitigating the risk of Long COVID fatigue. Enhancing the early support and treatment of patients with Long COVID, particularly for those who experienced “cognitive and mental symptoms” during acute infection, is crucial.

### Strengths and Limitations

Our study population consists of non-hospitalized COVID-19 patients with mild-to-moderate COVID-19 courses from the region of Augsburg. Therefore, transferring the results to patients with a severe disease course or to another population should be carried out with caution. As participants retrospectively reported their symptoms, recall bias cannot be excluded. Also, certain symptoms overlap with others, making it challenging to rule out the possibility of bias. Additionally, we do not know whether the persisting fatigue is favored by other comorbidities (excluding depression or anxiety disorder) or the intake of medication.

A strength of our work is that our analysis included a very homogeneous sample, in which each participant had to have a positive PCR test of SARS-CoV-2 infection, not relying on self-reports only. As we focused only on outpatients, the possible confounding factors introduced in severe and hospitalized patients (specific treatments or the development of other comorbidities) can be excluded. In addition, we cover a wide range of COVID-19 symptoms with the query of forty-two symptoms. Also, we used standardized questionnaires, which makes comparability with other results possible. The application of different methodical approaches underlined the consistency and robustness of results.

## 5. Conclusions

As Long COVID fatigue is a burden for the patients and their families, its prevention or most early detection and treatment is of high importance. Clinicians should consider that COVID symptoms may present differently depending on sex. With the herein identified specific symptom cluster that is strongly associated with Long COVID fatigue, clinicians may identify cases at risk earlier, thus enabling early therapeutic intervention. Additional research is needed to elucidate the underlying causes of Long COVID fatigue and identify suitable and timely preventive and therapeutic strategies for patients. These findings may also apply to other viral infections that lead to long-term health impairments, including fatigue. Finally, we would like to emphasize to continue collecting data on this issue, especially in outpatients, in order to strengthen the data.

## Figures and Tables

**Table 1:** Characteristics of the study sample, stratified by sex.

	N	Sex		*p*-Value ^1^
		Male, N = 205	Female, N = 245	
Age (years)	450			0.07
Median (IQR)		51.0 (36.0; 60.0)	48.0 (33.0; 57.0)	
Range		18.0; 87.0	18.0; 86.0	
Body mass index (kg/m^2^)	449			<0.001
Median (IQR)		26.2 (24.0; 29.0)	23.8 (21.5; 28.0)	
Range		17.5; 53.7	15.2; 49.6	
Missing		1	0	
Smoking status	450			0.005
Never smoked		91 (44.39%)	146 (59.59%)	
Ex-smoker		92 (44.88%)	78 (31.84%)	
Current smoker		22 (10.73%)	21 (8.57%)	
Time since acute infection (days)	450			0.84
Median (IQR)		244.0 (143.0; 322.0)	238.0 (123.0; 331.0)	
Range		32.0; 436.0	31.0; 441.0	
History of depression	449			0.07
Yes		12 (5.88%)	26 (10.61%)	
No		192 (94.12%)	219 (89.39%)	
Missing		1	0	
History of anxiety	448			0.43
Yes		9 (4.43%)	15 (6.12%)	
No		194 (95.57%)	230 (93.88%)	
Missing		2	0	
Fatigue	447			<0.001
No fatigue		152 (75.25%)	135 (55.10%)	
Fatigue		50 (24.75%)	110 (44.90%)	
Missing		3	0	
Fatigue Assessment Scale (FAS)—Total Score	447			<0.001
Median (IQR)		18.0 (15.0; 21.0)	20.0 (16.0; 26.0)	
Range		10.0; 43.0	10.0; 46.0	
Missing		3	0	
Symptom clusters				
Loss of sense: taste and/or smell	450			0.002
Loss of sense: taste and/or smell		124 (60.49%)	181 (73.88%)	
No loss of sense		81 (39.51%)	64 (26.12%)	
Locomotor system	450			0.13
Median (IQR)		1 (0.0; 2.0)	2 (1.0; 3.0)	
Gastrointestinal	450			<0.001
Median (IQR)		1 (0.0; 2.0)	1 (1.0; 2.0)	
Ear, nose and throat	450			0.22
Median (IQR)		4 (3.0; 6.0)	5 (3.0; 6.0)	
Cardiopulmonary	450			<0.001
Median (IQR)		1 (0.0; 2.0)	2 (0.0; 3.0)	
Cognitive and mental	450			<0.001
Median (IQR)		3 (1.0; 4.0)	3 (2.0; 5.0)	
Eyes/Hair/Skin/Stings in arms and legs	450			0.021
Median (IQR)		0 (0.0; 1.0)	0 (0.0; 1.0)	

^1^ Wilcoxon rank sum test; Pearson’s Chi-squared test.

**Table 2:** Clustering of forty-two acute COVID-19 symptoms.

	Symptom Clusters of Acute SARS-CoV-2 Infection						
	Loss of Sense: Taste and/or Smell	Ear, Nose and Throat	Cardiopulmonary	Cognitive and Mental	Locomotor System	Gastrointestinal	Eyes/Hair/Skin/Stings in Arms and Legs
Characteristic	Binary	Continuous	Continuous	Continuous	Continuous	Continuous	Continuous
Range	0;1 ^a^	0–10	0–5	0–8	0–5	0–6	0–6
Symptoms	Loss of sense of taste	Stuffy Nose	Shortness of breath at rest	Tiredness or exhaustion	Dizziness	Loss of appetite	Watering eyes
	Loss of sense of smell	Rhinitis or runny nose	Shortness of breath under exertion	Sleepiness	Problems with the coordination of body movements	Nausea or vomiting	Impaired vision
		Sore throat or pharyngeal pain	Feeling of pressure or pain in the chest	Concentration difficulties	Muscular weakness	Acid reflux	Red eyes or conjunctivitis
		Cough	Cyanotic lips	Sleep disorders	Muscular stiffness	Abdominal pain	Feeling of pinpricks in arms and legs
		Headache	Strong palpitations	Memory problems	Muscle or joint pain	Diarrhea	Loss of hair
		Hemoptysis		Depressed mood		Flatulence	Eczema
		Increased body temperature		Anxiety or panic			
		Fever		Mood swings			
		Chills and shivering					
		Pain when swallowing					

^a^ Either one of the two symptoms, or both were coded as 1.

**Table 3:** Log-linear regression model of the association between mutually adjusted symptom clusters and the FAS score (N = 446).

Characteristics ^1^	Percentage Change of the ß Estimate	ß Estimate	95% CI ^2^	*p*-Value	q-Value ^3^
Clustered symptoms: Loss of sense: taste and/or smell					
Yes ^4^	2.02	0.02	−0.04; 0.08	0.55	0.77
Clustered symptoms: Ear, nose and throat	0	0	−0.01; 0.02	0.66	0.77
Clustered symptoms: Cardiopulmonary	3.05	0.03	0.01; 0.06	**0.018**	0.08
Clustered symptoms: Cognitive and mental	5.13	0.05	0.04; 0.07	**<0.001**	**<0.001**
Clustered symptoms: Locomotor system	0	0	−0.03; 0.03	0.96	0.96
Clustered symptoms: Gastrointestinal	1.01	0.01	−0.02; 0.03	0.61	0.77
Clustered symptoms: Eyes/Hair/Skin/Stings in arms and legs	3.05	0.03	0.00; 0.07	0.08	0.16

^1^ Additionally adjusted for: age, sex, body mass index (kg/m^2^), smoking status, depression, anxiety. ^2^ CI = Confidence Interval. ^3^ False discovery rate corrected *p*-value. ^4^ Reference group: “No”.

**Table 4:** Logistic regression model of the association between mutually adjusted symptom clusters and fatigue (yes/no) as dependent variable (N = 446).

Characteristics ^1^	OR ^2^	95% CI ^3^	*p*-Value	q-Value ^4^
Clustered symptoms: Loss of sense: taste and/or smell				
Yes ^5^	1.43	0.85; 2.41	0.18	0.48
Clustered symptoms: Ear, nose and throat	1.02	0.90; 1.15	0.73	0.86
Clustered symptoms: Cardiopulmonary	1.22	0.99; 1.51	0.060	0.21
Clustered symptoms: Cognitive and mental	1.42	1.23; 1.66	**<0.001**	**<0.001**
Clustered symptoms: Locomotor system	0.99	0.79; 1.23	0.92	0.92
Clustered symptoms: Gastrointestinal	1.13	0.92; 1.40	0.24	0.48
Clustered symptoms: Eyes/Hair/Skin/Stings in arms and legs	1.10	0.82; 1.47	0.53	0.76

^1^ Adjusted for: age, sex, body mass index (kg/m^2^), smoking status, depression, anxiety. ^2^ OR = Odds Ratio, ^3^ CI = Confidence Interval. ^4^ False discovery rate corrected *p*-values. ^5^ Reference group: “No”.

## Data Availability

Data available on request due to restrictions (legal or ethical reasons).

## Supplementary Materials

The following supporting information can be downloaded at: https://www.mdpi.com/article/10.3390/jpm14060602/s1, Table S1: Log-linear regression analysis including only the male stratum: Association between symptom cluster (mutually adjusted) and the FAS-Score as dependent variable (N = 201), Table S2: Log-linear regression analysis including only the female stratum, of the association between symptom cluster (mutually adjusted) and the FAS-Score as dependent variable (N = 245), Table S3: Logistic regression analysis including only the male stratum, of the association between symptom clusters (mutually adjusted) and fatigue (yes/ no) as dependent variable (N = 201) and Table S4: Logistic regression analysis including only the female stratum, of the association between symptom clusters (mutually adjusted) and fatigue (yes/ no) as dependent variable (N = 245).

## List of Abbreviations

CFS	Chronic Fatigue Syndrome
ME	Myalgic encephalomyelitis
COVID-T	COVID thrombosis study
BMI	Body mass index
FAS	Fatigue Assessment Scale
VR-12	Validated Veterans RAND 12-Item Health Survey
MCS-12	Mental Component Summary Scale
PCS-12	Physical Component Summary Scale
IQR	Interquartile range
OR	Odds ratio
CI	Confidence interval
FDR	False discovery rate
