# Supplementary material for: Symptom Clusters in Acute SARS-CoV-2 Infection and Long COVID Fatigue in Male and Female Outpatients
Source: J Pers Med. 2024 Jun 5;14(6):602. doi: 10.3390/jpm14060602 (PMC11205233; doi:10.3390/jpm14060602)
Supplement: Supplementary file 1 [file jpm-14-00602-s001.zip › Supplementary Material S1.pdf]

Table S1 Log-linear regression analysis including only the male stratum: Association between symptom cluster (mutually adjusted) and the FAS-Score as dependent variable (n=201)

Characteristic	Change in Percent	95% CI[1]	p-value	q-value[2]
Clustered symptoms: Loss of sense: taste and/ or smell				
No	—	—		
Yes	-1.00	-0.09, 0.08	0.9	0.9
Clustered symptoms: Ear, nose and throat	0.00	-0.02, 0.03	0.7	0.8
Clustered symptoms: Cardiopulmonary	2.02	-0.03, 0.06	0.5	0.8
Clustered symptoms: Cognitive and mental	5.13	0.02, 0.08	**<0.001**	**0.009**
Clustered symptoms: Locomotor system	-1.00	-0.05, 0.03	0.5	0.8
Clustered symptoms: Gastrointestinal	3.05	-0.01, 0.08	0.12	0.5
Clustered symptoms: Eyes/ Hair/ Skin/ Stings in arms & legs	2.02	-0.03, 0.08	0.5	0.8
Age (years)	0.00	0.00, 0.00	0.4	0.8
Body mass index (kg/m²)	0.00	-0.01, 0.01	0.8	0.8
Smoker status				
Never smoked	—	—		
Ex-smoker	2.02	-0.06, 0.11	0.6	0.8
Current smoker	2.02	-0.11, 0.15	0.8	0.8
Prior diagnosis of depression disorder				
No	—	—		
Yes	7.25	-0.12, 0.26	0.5	0.8
Prior diagnosis of anxiety disorder				
No	—	—		
Yes	29.69	0.04, 0.49	**0.020**	0.13

[1]CI = Confidence Interval

[2]False discovery rate correction for multiple testing
