# Supplementary material for: Symptom Clusters in Acute SARS-CoV-2 Infection and Long COVID Fatigue in Male and Female Outpatients
Source: J Pers Med. 2024 Jun 5;14(6):602. doi: 10.3390/jpm14060602 (PMC11205233; doi:10.3390/jpm14060602)
Supplement: Supplementary file 1 [file jpm-14-00602-s001.zip › Supplementary Material S2.pdf]

Table S2 Log-linear regression analysis including only the female stratum, of the association between symptom cluster (mutually adjusted) and the FAS-Score as dependent variable (n=245)

| Characteristic | Change in Percent | 95% CI[1] | p-value | q-value[2] |
|---|---|---|---|---|
| Clustered symptoms: Loss of sense: taste and/ or smell | | | | |
| No | — | — | | |
| Yes | 5.13 | -0.04, 0.14 | 0.3 | 0.4 |
| Clustered symptoms: Ear, nose and throat | 0.00 | -0.02, 0.03 | 0.6 | 0.7 |
| Clustered symptoms: Cardiopulmonary | 3.05 | 0.00, 0.07 | **0.048** | 0.2 |
| Clustered symptoms: Cognitive and mental | 6.18 | 0.03, 0.08 | **<0.001** | **<0.001** |
| Clustered symptoms: Locomotor system | 1.01 | -0.03, 0.05 | 0.6 | 0.7 |
| Clustered symptoms: Gastrointestinal | -1.00 | -0.04, 0.03 | 0.7 | 0.7 |
| Clustered symptoms: Eyes/ Hair/ Skin/ Stings in arms & legs | 3.05 | -0.01, 0.08 | 0.2 | 0.3 |
| Age (years) | 0.00 | -0.01, 0.00 | 0.054 | 0.2 |
| Body mass index (kg/m²) | 1.01 | 0.00, 0.01 | 0.2 | 0.3 |
| Smoker status | | | | |
| Never smoked | — | — | | |
| Ex-smoker | 4.08 | -0.04, 0.13 | 0.3 | 0.5 |
| Current smoker | -1.98 | -0.15, 0.12 | 0.8 | 0.8 |
| Prior diagnosis of depression disorder | | | | |
| No | — | — | | |
| Yes | 12.75 | -0.01, 0.25 | 0.080 | 0.3 |
| Prior diagnosis of anxiety disorder | | | | |
| No | — | — | | |
| Yes | 12.75 | -0.04, 0.28 | 0.14 | 0.3 |

[1]CI = Confidence Interval

[2]False discovery rate correction for multiple testing
