# Supplementary material for: Symptom Clusters in Acute SARS-CoV-2 Infection and Long COVID Fatigue in Male and Female Outpatients
Source: J Pers Med. 2024 Jun 5;14(6):602. doi: 10.3390/jpm14060602 (PMC11205233; doi:10.3390/jpm14060602)
Supplement: Supplementary file 1 [file jpm-14-00602-s001.zip › Supplementary Material S3.pdf]

Table S3 Logistic regression analysis including only the male stratum, of the association between symptom clusters (mutually adjusted) and fatigue (yes/ no) as dependent variable (N=201)

Characteristic	OR[1]	95% CI[1]	p-value	q-value[2]
Clustered symptoms: Loss of sense: taste and/ or smell				
0	—	—		
1	0.99	0.43, 2.30	>0.9	>0.9
Clustered symptoms: Ear, nose and throat	1.05	0.86, 1.28	0.6	0.9
Clustered symptoms: Cardiopulmonary	1.08	0.73, 1.60	0.7	0.9
Clustered symptoms: Cognitive and mental	1.45	1.11, 1.92	**0.007**	0.085
Clustered symptoms: Locomotor system	0.84	0.59, 1.18	0.3	0.9
Clustered symptoms: Gastrointestinal	1.56	1.09, 2.27	**0.017**	0.11
Clustered symptoms: Eyes/ Hair/ Skin/ Stings in arms & legs	0.86	0.52, 1.41	0.6	0.9
Age (years)	1.00	0.98, 1.03	0.8	>0.9
Body mass index (kg/m²)	0.98	0.88, 1.07	0.6	0.9
Smoker status				
Never smoked	—	—		
Ex-smoker	0.74	0.33, 1.64	0.5	0.9
Current smoker	0.40	0.09, 1.51	0.2	0.7
Prior diagnosis of depression disorder				
No	—	—		
Yes	0.90	0.15, 4.66	>0.9	>0.9
Prior diagnosis of anxiety disorder				
No	—	—		
Yes	5.58	0.87, 41.1	0.073	0.3

[1]OR = Odds Ratio, CI = Confidence Interval

[2]False discovery rate correction for multiple testing
