# Supplementary material for: Symptom Clusters in Acute SARS-CoV-2 Infection and Long COVID Fatigue in Male and Female Outpatients
Source: J Pers Med. 2024 Jun 5;14(6):602. doi: 10.3390/jpm14060602 (PMC11205233; doi:10.3390/jpm14060602)
Supplement: Supplementary file 1 [file jpm-14-00602-s001.zip › Supplementary Material S4.pdf]

Table S4 Logistic regression analysis including only the female stratum, of the association between symptom clusters (mutually adjusted) and fatigue (yes/ no) as dependent variable (N=245)

Characteristic	OR[1]	95% CI[1]	p-value	q-value[2]
Clustered symptoms: Loss of sense: taste and/ or smell				
0	—	—		
1	2.05	1.02, 4.25	**0.049**	0.3
Clustered symptoms: Ear, nose and throat	1.01	0.85, 1.19	>0.9	>0.9
Clustered symptoms: Cardiopulmonary	1.21	0.93, 1.57	0.2	0.4
Clustered symptoms: Cognitive and mental	1.45	1.20, 1.76	**<0.001**	**0.002**
Clustered symptoms: Locomotor system	1.10	0.81, 1.50	0.5	0.7
Clustered symptoms: Gastrointestinal	0.95	0.72, 1.26	0.7	0.8
Clustered symptoms: Eyes/ Hair/ Skin/ Stings in arms & legs	1.30	0.88, 1.96	0.2	0.4
Age (years)	0.98	0.96, 1.01	0.2	0.4
Body mass index (kg/m²)	1.04	0.98, 1.10	0.2	0.4
Smoker status				
Never smoked	—	—		
Ex-smoker	1.49	0.75, 2.95	0.3	0.4
Current smoker	0.53	0.15, 1.65	0.3	0.4
Depression				
0	—	—		
1	1.27	0.43, 3.76	0.7	0.8
Anxiety				
0	—	—		
1	2.14	0.59, 8.08	0.2	0.4

[1]OR = Odds Ratio, CI = Confidence Interval

[2]False discovery rate correction for multiple testing
